# Association of Care at Minority-Serving vs Non–Minority-Serving Hospitals With Use of Palliative Care Among Racial/Ethnic Minorities With Metastatic Cancer in the United States

**DOI:** 10.1001/jamanetworkopen.2018.7633

**Published:** 2019-02-01

**Authors:** Alexander P. Cole, David-Dan Nguyen, Akezhan Meirkhanov, Mehra Golshan, Nelya Melnitchouk, Stuart R. Lipsitz, Kerry L. Kilbridge, Adam S. Kibel, Zara Cooper, Joel Weissman, Quoc-Dien Trinh

**Affiliations:** 1 Center for Surgery and Public Health, Brigham and Women’s Hospital, Harvard Medical School, Boston, Massachusetts; 2 Division of Urological Surgery, Brigham and Women’s Hospital, Harvard Medical School, Boston, Massachusetts; 3 Faculty of Medicine, McGill University, Montreal, Quebec, Canada; 4 Nazarbayev Intellectual School of Physics and Mathematics, Almaty, Kazakhstan; 5 Breast Oncology Program, Dana-Farber/Brigham and Women's Cancer Center, Boston, Massachusetts; 6 Department of Surgery, Brigham and Women's Hospital, Harvard Medical School, Boston, Massachusetts; 7 Division of General Internal Medicine, Brigham and Women’s Hospital, Harvard Medical School, Boston, Massachusetts; 8 Lank Center for Genitourinary Malignancies, Dana-Farber/Brigham and Women's Cancer Center, Boston, Massachusetts

## Abstract

**Importance:**

It is not known whether racial/ethnic differences in receipt of palliative care are attributable to different treatment of minorities or lower utilization of palliative care at the relatively small number of hospitals that treat a large portion of minority patients.

**Objective:**

To assess the association of receipt of palliative care among patients with metastatic cancer with receipt of treatment at minority-serving hospitals (MSHs) vs non-MSHs.

**Design, Setting, and Participants:**

This retrospective cohort study used Participant Use Files of the National Cancer Database, a prospectively maintained, hospital-based cancer registry consisting of all patients treated at more than 1500 US hospitals, to collect data from individuals older than 40 years with metastatic prostate, lung, colon, and breast cancer, diagnosed from January 1, 2004, to December 31, 2015. Data were accessed in October 2017, and the analysis was performed in July 2018.

**Exposures:**

Hospitals in the top decile in terms of the proportion of black and Hispanic patients for each cancer type were defined as MSHs.

**Main Outcomes and Measures:**

A multilevel logistic regression model that estimated the odds of palliative care was fit, adjusting for year of diagnosis, sex, race/ethnicity, insurance, income, educational level, and cancer type, with an interaction term between cancer type and MSH status and a hospital-level random intercept to account for unmeasured hospital characteristics.

**Results:**

A total of 601 680 individuals (mean [SD] age, 67.4 [11.4] years; 95% CI, 67.2-67.6 years; 314 279 [52.2%] male; 475 039 [78.9%] white) were studied. In total, 130 813 patients (21.7%) received palliative care, ranging from 102 019 (25.4%) with lung cancer to 9966 (11.1%) with colon cancer. In total, 16 435 black individuals (20.0%) and 3551 Hispanic individuals (15.9%) received palliative care vs 106 603 non-Hispanic white individuals (22.5%) (*P* < .001). The MSH patients were less likely than the non-MSH patients to receive palliative care, regardless of race/ethnicity (12 692 [18.0%] vs 118 121 [22.3%]; *P* = .002). In an adjusted analysis, treatment at an MSH had a statistically significant association with lower odds of receiving palliative care (odds ratio, 0.67; 95% CI, 0.53-0.84).

**Conclusions and Relevance:**

Although the factors associated with minority patients’ receipt of palliative care are complex, in this study, treatment at MSHs was associated with significantly lower odds of receiving any palliative care in an adjusted analysis, but black and Hispanic race/ethnicity was not. These findings suggest that the site of care is associated with race/ethnicity-based differences in palliative care.

## Key Points

**Question:**

Is receipt of treatment at minority-serving hospitals associated with lower use of palliative care among racial/ethnic minorities with cancer compared with non–minority-serving hospitals?

**Findings:**

In this cohort study of 601 680 individuals with metastatic prostate, lung, colon, and breast cancer in the United States, treatment at a minority-serving hospital had a statistically significant association with lower odds of receiving palliative care compared with treatment at a non–minority-serving hospital; patient race/ethnicity did not.

**Meaning:**

Site of care may represent a factor associated with minority patients’ lower odds of receiving palliative care.

## Teaser

This cohort study uses data from the National Cancer Database to assess the association between receipt of treatment at minority-serving vs non–minority-serving hospitals and use of palliative care among racial/ethnic minorities with cancer.

## Introduction

Palliative care plays a central role in the management of advanced cancer. Despite advances in targeted chemotherapy and immunotherapy, cancer remains the second leading cause of death in the United States,^1^ and most patients with metastatic cancer will ultimately die of their disease. For these patients, receipt of palliative care is associated with improved quality of life and prolonged survival.^2^

The presence of race/ethnicity-based disparities in health care and health outcomes is well documented. Racial/ethnic minorities often receive worse care and have worse outcomes.^3^ In cancer specifically, there are disparities in screening,^4^ treatment,^5,6^ and survival.^7^ Race/ethnicity-based differences have also been found in receipt of end-of-life care.^8,9^

Although much research on racial/ethnic differences in care has focused on patient characteristics^10^ and physician bias,^11,12^ there is an increasing effort to also investigate the role of the site of care.^13,14,15,16,17^ Because hospital care for most minority patients is concentrated at a comparatively small number of facilities,^18^ differences in care at these minority-serving hospitals (MSHs) could explain worse population-level outcomes for minorities overall. If so, policies to improve care at these hospitals represent a potential strategy to address race/ethnicity-based disparities.

We assessed racial/ethnic differences in receipt of palliative care for individuals diagnosed with metastatic prostate, lung, colon, and breast cancer. We examined whether receipt of palliative care differed by site of care and whether racial/ethnic disparities in receipt of palliative care are associated with minority patients receiving treatment in a subset of hospitals where palliative care is less often provided.

## Methods

### Data Source

The data for this study were abstracted from the Participant Use Files of the National Cancer Database (NCDB), a US cancer registry combining data on patients seen at any 1 of 1500 Commission on Cancer–accredited institutions in the United States.^19^ The NCDB registry is a joint project of the American Cancer Society and the Commission on Cancer of the American College of Surgeons, comprising more than 29 million unique cases. Trained data abstractors use standardized methods to collect sociodemographic and clinical data, including tumor type, stage, grade, and treatments.^20^ The NCDB captures 50.8% of all prostate cancers, 82.1% of all lung cancers, 62.5% of all colon cancers, and 66.6% of all breast cancers diagnosed in the United States.^21^ Data were accessed in October 2017, and the analysis was performed in July 2018. The study was approved by the Brigham and Women’s Hospital Institutional Review Board under a general study protocol for analyses using NCDB data, which included a waiver of informed consent because the information in the Commission on Cancer’s NCDB is deidentified. This study conformed to the Strengthening the Reporting of Observational Studies in Epidemiology (STROBE) reporting guideline for reporting observational research.^22^

### Study Cohort

We chose to focus on men and women 40 years and older with metastatic prostate, non–small cell lung, colon, and breast cancer. These 4 cancer types were chosen because they represented the most common and most lethal cancers for men and women during the study period.^23^ We chose individuals diagnosed with each cancer from January 1, 2004, to December 31, 2015, using the following *International Classification of Diseases for Oncology, Third Edition* topography codes: prostate C619, lung C340 to C349, colon C180 to C189 and C260, and breast C500 to C509. We selected men and women with confirmed distant metastases based on the American Joint Committee on Cancer staging system.^24^ We excluded individuals who had missing follow-up information as well as those diagnosed when younger than 40 years because facility information on these patients is censored by the NCDB for confidentiality purposes.

### Receipt of Palliative Care

The main outcome measure was receipt of any palliative care services. Receipt of palliative care is a variable included with the Participant Use Files of the NCDB. Receipt of palliative care is determined by NCDB data abstractors based on patients’ clinical medical records at participating institutions. Treatments are coded as palliative only if it is explicitly mentioned that the goal of treatment is palliation and not cure (eg, pain control after a routine surgical procedure would not be coded as palliative care). Palliative care encompasses surgical treatment, radiation therapy, and systemic chemotherapy administered to alleviate symptoms but not to cure.^25^ For the purposes of this study, palliative care was treated as a dichotomous variable.

### MSH Status

The site of care was the facility reporting the case to the NCDB. This facility is typically the site of diagnosis. For those who received care at multiple institutions, the site of care was the facility where they received definitive cancer care. The MSH status was calculated for each facility based on the proportion of minority patients as follows. First, hospitals were ranked in terms of the proportion of minority patients (black or Hispanic). Second, we identified hospitals in the top decile when ranked from least to greatest proportion of minority patients.^26,27^ Hospitals in the top decile were considered MSHs. We used the entire population with a diagnosis, not limiting to metastatic cancer only (eg, number of black and Hispanic men with prostate cancer [any stage] at an institution as a portion of the total number of men with prostate cancer [any stage] at that institution and so forth).

### Covariates

Baseline sociodemographic covariates included age at diagnosis, sex, race/ethnicity (white non-Hispanic, black non-Hispanic [henceforth referred to as white and black], Hispanic, Asian, other, or unknown), and year of diagnosis. Sociodemographic variables include primary insurance carrier (private, Medicaid or other government payer, Medicare, uninsured, and unknown), educational level (estimated from the percentage of adults within the patient’s zip code without a high school diploma [<7%, 7%-12.9%, 13%-20.9%, or ≥21%]), and zip code–level median household income (<$38 000, $38 000-$47 999, $48 000-$62 999, or ≥$63 000). Clinical covariates included clinical comorbidity (based on the Charlson-Deyo Comorbidity Index, categorized into 0, 1, or ≥2) and cancer type. Because all patients in the cohort had distant metastases (stage IV), we did not adjust by clinical stage. Facility caseload was defined for each cancer as the mean of the total volume of patients with any stage disease treated at the facility for each cancer type in the year of the patient’s diagnosis. This calculation was performed using a previously defined method for NCDB data to account for some facilities leaving and entering the NCDB during the study.^28^

### Statistical Analysis

For each of the 4 cancer types, clinical covariates were compared between patients treated at MSHs and non-MSHs. Clustering was performed at the level of the facility to account for correlation of patient characteristics within hospitals. Means (SDs) were calculated for all continuous variables and proportions for all categorical variables. Given less than 5% of missing data in variables, missing values for covariates were ignored because this has a low probability of skewing results.^29^ Missing outcome variables (unknown whether palliative care was performed) were assumed to be nonignorable, and a maximum likelihood technique for our multilevel model was used to address this.^30^ We used χ^2^ tests with a Rao-Scott adjustment to account for clustering to compare the distribution of covariates between patients treated at MSHs and non-MSHs.^31,32^ We then performed a univariate analysis, again clustering by facility, to compare the proportion of patients receiving palliative care based on race/ethnicity and other baseline characteristics (eg, site of care, cancer type).

To assess the association among site of care, patient characteristics, cancer type, and palliative care, a multilevel logistic regression model was fit using the entire study population. This model included fixed-effect terms for patient clinical and demographic covariates (including race/ethnicity and cancer type) and site of care (MSH vs non-MSH). We included an interaction term between cancer type and MSH status to assess whether the effect of MSHs differed in a statistically significant fashion among the 4 cancer types. A facility-level random intercept was included to account for unmeasured hospital-level characteristics that might cut across multiple cancers.^33^ For example, some hospitals may have palliative care departments, whereas others may not.

Finally, based on a significant interaction term (between MSH and cancer type), we performed subgroup analyses by cancer type. For each cancer type, we fit separate multilevel models that assessed the association of clinical and demographic variables as well as site of care on the odds of receiving palliative care.

 All analyses were performed with Stata statistical software, version 14.0 (StataCorp). Statistical significance was defined as a 2-sided *P* < .05.

## Results

The study cohort consisted of 601 680 individuals (mean [SD] age, 67.4 [11.4] years; 95% CI, 67.2-67.6 years; 314 279 [52.2%] male; 475 039 [78.9%] white) with metastatic cancer diagnosed from January 1, 2004, to December 31, 2015. There were 44 521 men with metastatic prostate cancer, of whom 7096 (15.9%) were treated at MSHs. There were 402 912 men and women with metastatic non–small cell lung cancer, of whom 43 882 (9.4%) were treated at MSHs. There were 89 826 men and women with metastatic colon cancer, of whom 10 570 (11.8%) were treated at MSHs. Finally, of the 65 380 women and men with metastatic breast cancer, 9166 (14.0%) were treated at MSHs.

For all 4 cancer types, those treated at MSHs had lower educational levels, had lower income, and were less likely to have public insurance. The baseline characteristics of men and women treated for each of the 4 cancer types at MSHs and non-MSHs are summarized in Table 1.

**Table 1.** Baseline Characteristics of Patients With Metastatic Prostate, Lung, Colon, and Breast Cancer in the National Cancer Database

Characteristic	No. (%) of Patients											
	Prostate Cancer			Non–Small Cell Lung Cancer			Colon Cancer			Breast Cancer		
	MSHs	Non-MSHs	*P* Value^a^	MSHs	Non-MSHs	*P* Value^a^	MSHs	Non-MSHs	*P* Value^a^	MSHs	Non-MSHs	*P* Value^a^
Total patients	7095 (15.9)	37 426 (84.1)	NA	43 882 (9.4)	359 030 (90.6)	NA	10 570 (11.8)	79 256 (88.2)	NA	9166 (14.0)	56 214 (86.0)	NA
Palliative care												
Yes	831 (11.7)	5962 (16.0)	<.001	9452 (21.5)	92567 (25.8)	.02	1036 (9.8)	8930 (11.3)	.17	1373 (15.0)	10662 (19.0)	.003
No	6263 (88.3)	31405 (84.0)		34420 (78.5)	265841 (74.2)		9532 (90.2)	70260 (88.7)		7792 (85.0)	45354 (81.0)	
Sex												
Male	NA	NA	NA	25 320 (57.7)	198 359 (55.2)	<.001	5323 (50.4)	39 810 (50.2)	.84	150 (1.6)	796 (1.4)	.11
Female	NA	NA		18 562 (42.3)	160 671 (44.8)		5247 (49.6)	39 446 (49.8)		9016 (98.4)	55 418 (98.6)	
Age group, y												
40-50	289 (4.1)	1203 (3.2)	<.001	4080 (9.3)	24 620 (6.9)	<.001	1432 (13.5)	8682 (11.0)	<.001	1908 (20.8)	8648 (15.4)	<.001
51-60	1572 (22.2)	5628 (15.0)		11 282 (25.7)	71 508 (19.9)		2802 (26.5)	16 202 (20.4)		2816 (30.7)	14 939 (26.6)	
61-70	2227 (31.4)	9943 (26.6)		13 502 (30.8)	110 694 (30.8)		2818 (26.7)	19 763 (24.9)		2229 (24.3)	14 866 (26.4)	
71-80	1780 (25.1)	10 590 (28.3)		10 549 (24.0)	104 514 (29.1)		2047 (19.4)	18 839 (23.8)		1361 (14.8)	10 465 (18.6)	
≥81	1227 (17.3)	10 062 (26.9)		4469 (10.2)	47 694 (13.3)		1471 (13.9)	15 770 (19.9)		852 (9.4)	7296 (13.0)	
Race/ethnicity												
White	2137 (30.1)	29 250 (78.2)	<.001	20 542 (46.8)	306 885 (85.5)	<.001	3955 (37.4)	63 648 (80.3)	<.001	3368 (36.8)	45 254 (80.5)	<.001
Black	3463 (48.8)	5244 (14.0)		16 977 (38.7)	31 507 (8.8)		4425 (41.9)	9630 (12.2)		3916 (42.7)	7100 (12.6)	
Hispanic	1196 (16.9)	1538 (4.1)		4255 (9.7)	7865 (2.2)		1727 (16.3)	2723 (3.4)		1407 (15.4)	1728 (3.1)	
Asian	164 (2.3)	750 (2.0)		1408 (3.2)	8206 (2.3)		306 (2.9)	2017 (2.5)		280 (3.1)	1231 (2.2)	
Other	61 (0.9)	281 (0.7)		287 (0.7)	1929 (0.5)		83 (0.8)	549 (0.7)		76 (0.8)	377 (0.7)	
Unknown	74 (1.0)	363 (1.0)		413 (0.9)	2638 (0.7)		74 (0.7)	689 (0.9)		119 (1.3)	524 (0.9)	
Year of diagnosis												
2004-2006	1717 (24.2)	8350 (22.3)	.007	10 716 (24.4)	85 646 (23.9)	.30	2081 (19.7)	14 583 (18.4)	.19	2027 (22.1)	11 422 (20.3)	.02
2007-2009	2048 (28.9)	10 389 (27.8)		12 390 (28.2)	102 357 (28.5)		3101 (29.3)	23 001 (29.0)		2724 (29.7)	16 393 (29.2)	
2010-2012	2376 (33.5)	13 172 (35.2)		15 596 (35.5)	126 400 (35.2)		3985 (37.7)	30 695 (38.7)		3262 (35.6)	20 910 (37.2)	
2013-2015	954 (13.4)	5515 (14.7)		5180 (11.9)	44 627 (12.4)		1403 (28.7)	10 977 (13.9)		1153 (12.6)	7489 (13.3)	
Charlson-Deyo Comorbidity Index												
0	5448 (76.8)	28 556 (76.3)	.81	28 493 (64.9)	224 356 (62.5)	.10	7780 (73.6)	56 907 (71.8)	.20	7408 (80.8)	44 836 (79.7)	.25
1	1129 (15.9)	6075 (16.2)		10 524 (24.0)	92 953 (25.9)		2054 (19.4)	16 161 (20.4)		1320 (14.4)	8280 (14.8)	
≥2	518 (7.3)	2795 (7.5)		4865 (11.1)	41 721 (11.6)		736 (7.0)	6188 (7.8)		438 (4.8)	3098 (5.5)	
Insurance												
Private	1381 (19.5)	9298 (24.8)	<.001	10 721 (24.4)	105 008 (29.3)	<.001	2883 (27.3)	26 823 (33.8)	<.001	2752 (30.0)	22 142 (39.4)	<.001
Medicare	3335 (47.0)	23 539 (62.9)		20 846 (47.5)	205 995 (57.4)		4423 (41.8)	42 910 (54.1)		3061 (33.4)	24 636 (43.8)	
Medicaid	1011 (14.2)	1920 (5.1)		5928 (13.5)	22 778 (6.3)		1423 (13.5)	4624 (5.8)		1763 (19.2)	5175 (9.2)	
Other governmental	57 (0.8)	423 (1.1)		507 (1.2)	4774 (1.3)		68 (0.6)	654 (0.8)		59 (0.7)	419 (0.7)	
None	1011 (14.2)	1518 (4.1)		4328 (9.9)	14 125 (3.9)		1372 (13.0)	3057 (3.9)		1108 (12.1)	2702 (4.8)	
Unknown	300 (4.2)	728 (2.0)		1552 (3.5)	6350 (1.8)		401 (3.8)	1188 (1.5)		423 (4.6)	1140 (2.0)	
Family income, $^b^												
>63 000	1203 (17.0)	11 159 (29.8)	<.001	7252 (16.5)	98 561 (27.4)	<.001	1861 (17.6)	24 521 (30.9)	<.001	1662 (18.1)	18 222 (32.4)	<.001
49 000-63 000	1471 (20.7)	10 069 (26.9)		9047 (20.6)	95 824 (26.7)		2152 (20.4)	20 713 (26.1)		2003 (21.9)	14 918 (26.5)	
38 000-48 999	1498 (21.1)	8887 (23.7)		9492 (21.7)	90 470 (25.2)		2381 (22.5)	18 684 (23.6)		1996 (21.8)	12 729 (22.7)	
<38 000	2830 (39.9)	6679 (17.9)		17160 (39.1)	65337 (18.2)		3976 (37.6)	13 643 (17.2)		3357 (36.6)	9340 (16.6)	
Unknown	93 (1.3)	632 (1.7)		931 (2.1)	8838 (2.5)		200 (1.9)	1695 (2.2)		148 (1.6)	1005 (1.8)	
Educational level, % without high school diploma^b^												
Unknown	89 (1.3)	601 (1.6)	<.001	910 (2.1)	8645 (2.4)	<.001	198 (1.9)	1654 (2.1)	<.001	144 (1.6)	973 (1.7)	<.001
<7	595 (8.4)	9018 (24.1)		4024 (9.2)	73 948 (20.6)		1032 (9.8)	17 938 (22.6)		934 (10.2)	13 331 (23.7)	
7-12.9	1211 (17.0)	12 328 (32.9)		8787 (20.0)	119 752 (33.4)		2068 (19.6)	25 854 (32.6)		1756 (19.2)	18 982 (33.8)	
13-20.9	2108 (29.7)	9515 (25.5)		13 629 (31.0)	98 698 (27.5)		3241 (30.6)	21 094 (26.6)		2865 (31.3)	14 218 (25.3)	
>30	3092 (43.6)	5964 (15.9)		16 532 (37.7)	57 987 (16.2)		4031 (38.1)	12 716 (16.1)		3467 (37.8)	8710 (15.5)	

Abbreviations: MSH, minority-serving hospital; NA, not applicable.

^a^ Hospital-level clustering with Taylor series linearization; the Pearson χ^2^ test was used to test significance.

^b^ Both estimated using patients’ county of residence.

In the combined cohort, 130 813 patients (21.7%) received any palliative care and 470 867 (78.1%) did not. The number of patients receiving palliative care differed based on cancer type. The number of patients receiving palliative care was 6793 (15.3%) of those with metastatic prostate cancer, 102 019 (25.4%) of those with metastatic lung cancer, 9966 (11.1%) of those with metastatic colon cancer, and 120 035 (18.5%) of those with metastatic breast cancer (*P* < .001). In terms of race/ethnicity, whereas 106 603 white patients (22.5%) received palliative care, only 16 435 black patients (20.0%) and 3551 Hispanic patients (15.9%) received palliative care (*P* < .001 for all). Patients treated at an MSH were less likely than patients treated at a non-MSH to receive palliative care regardless of race/ethnicity (12 692 [18.0%] vs 118 121 [22.3%], *P* = .002). Receipt of palliative care based on other baseline characteristics is summarized in Table 2.

**Table 2.** Unadjusted Proportions of Patients With Metastatic Cancer Receiving Palliative Care in Overall Cohort by Baseline Characteristics

Characteristic	No. (%) of Patients		*P* Value^a^
	No Palliative Care	Any Palliative Care	
Total patients	470 867 (78.1)	130 813 (21.7)	NA
Hospital type			
MSH	58 007 (82.1)	12 692 (18.0)	.002
Non-MSH	412 860 (77.8)	118 121 (22.3)	
Cancer type			
Prostate	37 668 (84.7)	6793 (15.3)	<.001
Non–small cell lung	300 261 (74.6)	102 019 (25.4)	
Colon	79 792 (88.9)	9966 (11.1)	
Breast	53 146 (81.5)	12 035 (18.5)	
Sex			
Male	244 246 (77.8)	69 571 (22.2)	<.001
Female	226 621 (78.7)	61 242 (21.3)	
Age group, y			
≤50	39 413 (77.7)	11 305 (22.3)	<.001
51-60	97 147 (76.8)	29 331 (23.2)	
61-70	136 217 (77.5)	39 545 (22.5)	
71-80	125 971 (78.8)	33 977 (21.2)	
≥81	72 119 (81.2)	16 655 (18.8)	
Race/ethnicity			
White	367 695 (77.5)	106 603 (22.5)	<.001
Black	65 716 (80.0)	16 435 (20.0)	
Hispanic	18 814 (84.1)	3551 (15.9)	
Asian	11 782 (82.1)	2572 (17.9)	
Other	2879 (79.5)	741 (20.5)	
Unknown	3981 (81.4)	911 (18.6)	
Year of diagnosis			
2004-2006	108 557 (79.7)	27 602 (20.3)	<.001
2007-2009	135 234 (78.6)	36 847 (21.4)	
2010-2012	168 279 (77.8)	47 907 (22.2)	
2013-2015	58 797 (76.1)	18 457 (23.9)	
Charlson-Deyo Comorbidity Index			
0	318 898 (79.1)	84 038 (20.9)	<.001
1	105 984 (76.6)	32 434 (23.4)	
≥2	45 985 (76.2)	14 341 (23.8)	
Insurance			
Private	141 937 (78.5)	38 986 (21.6)	<.001
Medicare	257 850 (78.5)	70 705 (21.5)	
Medicaid	33 980 (76.2)	10 624 (23.8)	
Other governmental	5143 (73.9)	1816 (26.1)	
None	22 424 (76.8)	6784 (23.2)	
Unknown	9533 (83.4)	1898 (23.4)	
Family income, $^a^			
>63 000	130 096 (79.2)	34 101 (20.8)	.02
49 000-63 000	122 089 (78.3)	33 879 (21.7)	
38 000-48 999	113 059 (77.5)	32 849 (22.5)	
<38 000	95 269 (78.0)	26 824 (22.0)	
Unknown	10 354 (76.6)	3160 (16.6)	
Educational level, % without high school diploma^a^			
<7	94 022 (77.9)	26 614 (22.1)	<.001
7-12.9	147 688 (77.5)	42 865 (22.5)	
13-20.9	129 068 (78.2)	36 018 (21.8)	
>30	89 998 (80.2)	22 220 (19.8)	
Unknown	10 091 (76.5)	3096 (23.5)	

Abbreviations: MSH, minority-serving hospital; NA, not applicable.

^a^ Estimated from patients’ county of residence.

In our adjusted multilevel logistic regression model adjusting for age, race/ethnicity, comorbidity, cancer type, and patient demographics and including an interaction term between MSH status and cancer type, patients who received care at an MSH had two-thirds the odds of receiving palliative care compared with those who received care at a non-MSH (odds ratio [OR], 0.67; 95% CI, 0.53-0.84). Later study year was also associated with increased odds of receiving palliative care (first vs last period: OR, 1.30; 95% CI, 1.27-1.33). Patients with Medicaid and uninsured patients were more likely to receive palliative care compared with those with private insurance (Medicaid vs private: OR, 1.16 [95% CI, 1.13-1.19]; uninsured vs private: OR, 1.17 [95% CI, 1.13-1.21]).

After adjusting for MSH status and other covariates, the difference in receipt of palliative care between white and black individuals was no longer statistically significant (OR, 1.02; 95% CI, 0.99-1.04). Hispanic patients had higher odds of palliative care compared with white patients (OR, 1.06; 95% CI, 1.01-1.10). Compared with non-Hispanic white patients, a lower proportion of Asian patients received palliative care (2572 [17.9%] vs 106 603 [22.5%], *P* < .001). This finding was also true on adjusted analyses (OR, 0.93; 95% CI, 0.88-0.98). Table 3 provides a summary of the adjusted analyses.

**Table 3.** Factors Associated With Palliative Care in an Adjusted Multilevel Model Including a Hospital-Level Random Intercept

Indicator	Odds Ratio (95% CI)	*P* Value^a^
Hospital type		
Non-MSH	1 [Reference]	NA
MSH	0.67 (0.53-0.84)	.001
Metastatic cancer type		
Prostate	1 [Reference]	NA
Non–small cell lung	1.69 (1.51-1.88)	<.001
Colon	0.56 (0.50-0.63)	<.001
Breast	1.10 (0.98-1.23)	.08
Sex		
Male	1 [Reference]	NA
Female	0.95 (0.94-0.97)	<.001
Age group, y		
≤50	1 [Reference]	NA
51-60	1.00 (0.97-1.03)	.89
61-70	0.93 (0.90-0.95)	<.001
71-80	0.86 (0.83-0.88)	<.001
≥81	0.79 (0.77-0.82)	<.001
Race/ethnicity		
White	1 [Reference]	NA
Black non-Hispanic	1.02 (0.99-1.04)	.19
Hispanic	1.06 (1.01-1.10)	.01
Asian	0.93 (0.88-0.98)	.008
Other	0.92 (0.85-1.01)	.08
Unknown	0.78 (0.72-0.84)	<.001
Year of diagnosis		
2004-2006	1 [Reference]	NA
2007-2009	1.10 (1.08-1.12)	<.001
2010-2012	1.16 (1.14-1.18)	<.001
2013-2015	1.30 (1.27-1.33)	<.001
Charlson-Deyo Comorbidity Index		
0	1 [Reference]	NA
1	1.01 (0.99-1.03)	.18
≥2	1.00 (0.98-1.03)	.70
Insurance		
Private	1 [Reference]	NA
Medicare	1.01 (0.99-1.03)	.14
Medicaid	1.16 (1.13-1.19)	<.001
Other governmental	1.20 (1.13-1.27)	<.001
None	1.17 (1.13-1.21)	<.001
Unknown	0.87 (0.82-0.92)	<.001
Family income, $^a^		
>63 000	1 [Reference]	NA
49 000-63 000	0.99 (0.97-1.01)	.39
38 000-48 999	0.97 (0.95-1.00)	.06
<38 000	0.99 (0.96-1.02)	.46
Unknown	0.87 (0.65-1.16)	.34
Educational level, % without high school diploma^a^		
>30	1 [Reference]	NA
13-20.9	1.00 (0.98-1.02)	.94
7-12.9	1.00 (0.97-1.03)	.99
<7	1.00 (0.97-1.03)	.98
Unknown	1.24 (0.92-1.66)	.16

Abbreviations: MSH, minority-serving hospital; NA, not applicable.

^a^ Estimated from county of residence.

The interaction term between cancer type and MSH status was associated with receipt of palliative care. Thus, we performed a subgroup analysis stratifying by cancer type. In the metastatic prostate cancer subgroup, the odds of receiving palliative care at MSHs were approximately 33% lower (OR, 0.67; 95% CI, 0.55-0.82); in the lung cancer subgroup, the odds of palliative care were 27% lower at MSHs (OR, 0.73; 95% CI, 0.57-0.93); in colon cancer, the odds of palliative care at MSHs were not significantly lower (OR, 0.86; 95% CI, 0.67-1.09); and in breast cancer, the odds of palliative care were 27% lower (OR, 0.73; 95% CI, 0.59-0.89). As in the combined cohort, adjustment for MSH status in all cancers attenuated the association between race/ethnicity and odds of receiving palliative care toward the null.

## Discussion

In this retrospective, registry-based study of adults diagnosed with metastatic prostate, lung, breast, and colon cancer, there were significantly lower odds of receiving palliative care among patients treated at MSHs compared with non-MSHs. Although it has been previously reported that minority patients are less likely to receive palliative care services at the end of life,^8,9^ the present findings suggest that site of care may be a significant factor associated with race/ethnicity-based differences in palliative care.

The policy implications of this finding are significant. Given that care for minority patients is concentrated at a comparatively small number of hospitals in the United States, it is likely that one important strategy to address racial/ethnic disparities in palliative care is to focus on improving access to palliative care at the small number of hospitals that care for most minority patients. If initiatives to target palliative care use at MSHs are successful, national disparities in palliative care may be reduced.

Overall, this fits with an increasing understanding that the site of care is a determinant of health outcomes for minority patients. Although there are data that physicians may systematically treat black and white patients differently,^11,12^ that minority patients tend to receive care at different facilities is also important. More than being a function of individual behavior, there is increasing recognition that disparities in outcomes depend on different treatment of white and minority patients within the same hospital and systemic differences in where minority patients receive care.^14,15^

A previous study^18^ found that MSHs have higher readmission rates and worse performance in many clinical scenarios, for example, when treating acute myocardial infarctions and pneumonia. A study^34^ of emergency general surgery at MSHs found that hospital-level factors accounted for approximately 40% of increased odds for readmission, and inpatient mortality was significantly greater. Hospital leadership can also play an important role. A survey of chairmen at black-serving hospitals found that, when compared with non–black-serving hospital boards, these chairpersons report less expertise with quality-of-care issues and are less likely to give high priority to quality of care.^35^ An analysis^36^ of racial disparity in surgical mortality found that although gaps between black and white patients have narrowed overall, improvements were less likely among hospitals that served the highest proportion of minority patients. Overall, our findings suggest that similar systemic differences between MSHs and non-MSHs may be associated with the differences in receipt of palliative care among underserved minority patients.

Although Asian patients composed a small proportion of our population, they were less likely to receive palliative care after adjusting for MSH status. Asian individuals are a heterogeneous group and may in some cases have better access to health care compared with Hispanic patients and black patients; Asian individuals have population-level health outcomes that exceed most of the other racial/ethnic groups.^37^ Thus, as has been done in a prior study,^27^ we did not include Asian patients in our definition of MSHs. The lower odds of palliative care among Asian patients could reflect cultural differences, differences in familial characteristics among this population, and other economic or health systems factors.

The finding that palliative care is more common in Medicaid patients and uninsured patients was similarly surprising given that these patients seem to receive worse care based on many other health metrics.^38^ Perhaps these patients were presenting at a more advanced stage of disease, when palliative care is the only good option. Alternatively, perhaps the absence of a strong fee-for-service incentive toward doing more reduced the barrier for palliative care for the Medicaid and uninsured patients.

### Strengths and Limitations

Strengths of our study include our use of a large, accurate national registry, which captures most US patients diagnosed with 4 highly prevalent types of cancers. Another strength is that our study included patients from all payers. Our work therefore improves on earlier definitions of *minority serving*, which often used Medicare claims and therefore involved only the proportion of Medicare beneficiaries who were racial/ethnic minorities not the proportion of patients with a given condition.^26^

Despite these strengths, this work has limitations. Data on palliative care services are of uncertain accuracy. The data on receipt of palliative care in the NCDB were collected from medical records by trained data abstractors at each institution. Intent must be inferred from clinical records. Although we believe that record review may be more accurate than insurance claims, which have been reported to often have only moderate accuracy for ascertaining the intensity of end-of-life care,^39^ the accuracy may be lower than some prospective trials that have specifically assigned patients to palliative care interventions.^2^ Additional studies that specifically address interrater variability and validate this variable against other end points (eg, inappropriately aggressive end-of-life care, such as chemotherapy in the last 14 days of life, death in hospital, or death in the intensive care unit) would be useful. Another limitation is the possibility of unmeasured patient confounders, which are always a factor in retrospective research. Our use of a multilevel model with a hospital-level random intercept should account for unmeasured hospital characteristics at the level of the hospital (eg, some hospitals may have palliative care departments, whereas others may not).

Although the NCDB captures most patients with each of these 4 cancer types in the United States, data are not population based. Thus, certain patients who did not receive care at Commission on Cancer–accredited US hospitals may have been underrepresented. For example, if the database underrepresents poor-performing, rural non-MSHs, the disparities among MSHs could be inflated.

## Conclusions

These findings suggest that there are significant racial/ethnic disparities in receipt of palliative care for metastatic cancer within a large cohort of US patients with cancer. After controlling for race/ethnicity and MSH status, we found that treatment at MSHs was associated with significantly lower odds of receiving palliative care, but black and Hispanic race/ethnicity was not. Strategies that focus on improving palliative care use at MSHs may be an effective strategy to increase the receipt of palliative care for this population.
